# Reproductive life history of sablefish (*Anoplopoma fimbria*) from the U.S. Washington coast

**DOI:** 10.1371/journal.pone.0184413

**Published:** 2017-09-08

**Authors:** José M. Guzmán, J. Adam Luckenbach, Mollie A. Middleton, Kenneth C. Massee, Cortney Jensen, Frederick W. Goetz, Andrew J. Jasonowicz, Penny Swanson

**Affiliations:** 1 Environmental and Fisheries Sciences Division, Northwest Fisheries Science Center, National Marine Fisheries Service, NOAA, Seattle, WA, United States of America; 2 School of Aquatic and Fishery Sciences, University of Washington, Seattle, WA, United States of America; 3 Center for Reproductive Biology, Washington State University, Pullman, WA, United States of America; 4 Ocean Associates Inc., Under Contract to Northwest Fisheries Science Center, National Marine Fisheries Service, NOAA, Seattle, WA, United States of America; Universitat de Barcelona, SPAIN

## Abstract

Sablefish (*Anoplopoma fimbria*) is a marine groundfish that supports valuable fisheries in the North Pacific Ocean and holds promise for marine aquaculture. Limited information is available, however, about its reproductive biology. This study aimed to characterize the complete reproductive cycle, including seasonal changes in gonadal development (macroscopic and histological), plasma sex steroid levels (17β-estradiol -E2-, and 11-ketotestosterone -11KT-), gonadosomatic and hepatosomatic indices (GSI, and HSI), and condition factor (K) of female and male sablefish captured off the Washington coast. Adult fish (209 females, 159 males) were caught by longline monthly from August 2012 to August 2013. Early signs of recruitment of ovarian follicles into secondary growth, indicated by oocytes containing small yolk granules and cortical alveoli, were first observed in March. Oogenesis progressed during spring and summer, and fully vitellogenic follicles were first observed in July. Vitellogenic growth was correlated with increases in plasma E2, GSI, HSI and K. Periovulatory females, indicated by fully-grown oocytes with migrating germinal vesicles and hydrated oocytes, were found from November to February. At this stage, plasma E2 and GSI reached maximal levels. In males, proliferating cysts containing spermatocytes were first observed in April. Testicular development proceeded during spring and summer, a period during which all types of male germ cells were found. The first clusters of spermatozoa appeared in July, concomitant with a 5.2-fold increase in GSI. Spermiating males were observed from November to April; at this time, spermatids were absent or greatly reduced, and testis lobules were filled with spermatozoa. The highest levels of plasma 11KT were found in males at this stage. Postspawning ovaries and testes, and basal steroids levels were found in fish captured from February to April. These results suggest that sablefish in coastal Washington initiate their reproductive cycle in March/April and spawn primarily in January/February.

## Introduction

Sablefish (*Anoplopoma fimbria*, order Scorpaeniformes), also known as black cod, is a groundfish native to the North Pacific Ocean ranging from Baja California to the Bering sea, and throughout the Aleutian Islands into waters off the Kamchatka Peninsula, Russia and northern Japan. Sablefish supports valuable fisheries in Alaska, Japan, Russia, and along the U.S. West Coast, where it is the target of restricted commercial and recreational fisheries [1–2]. In addition, based on its high growth rate and market value, sablefish is a developing marine aquaculture species in several countries, including the U.S. and Canada.

A number of stock assessment reports have provided valuable information on the reproductive biology of sablefish since the early 1980s, including size and age at sexual maturity, fecundity estimates and spawning season along the northeast Pacific coast. Sablefish exhibit sexual dimorphism, with females growing larger than males [3–4]. Length at fifty percent maturity is approximately 55 cm for females and 51 cm for males in coastal waters of Washington, Oregon and northern California, which is attained at an age of approximately 5 years [5–7]. Sablefish spawn in winter, from January through March along the U.S. West Coast [8–9], with a peak in February [5]. While spawning may occur as shallow as 300 m [8], Hunter and colleagues (1989) found the majority of actively spawning females at 800 m or deeper. The annual fecundity of sablefish appears to be determinate, in which the clutch of advanced fully-yolked oocytes before spawning is equivalent to the potential annual fecundity [9–10]. A recent study suggested that up to 21% of female sablefish captured in the Gulf of Alaska in December would skip spawning that season [11]; however, there are no reports of skipped spawning in sablefish from lower latitudes.

Head et al. [6] described stages of oogenesis at the histological level in females captured by bottom trawl in waters from the U.S.-Canada to the U.S.-Mexico border at two time periods (May-July and August-October). This study provided information on the histological appearance of the ovary during early development and on reproduction in sablefish; however, there are a number of important aspects of the reproductive biology of this species that remain unstudied. For example, while the spawning time has been defined for specific areas of the North Pacific Ocean, little is known about the chronology of sablefish oogenesis during a complete reproductive cycle, including the onset of vitellogenesis. With regard to males, information on spermatogenesis is limited to anecdotal findings of running ripe, pre-spawning specimens [9, 12]. Furthermore, endocrine changes during gametogenesis have not been explored in this species for either sex.

Gametogenesis in teleosts is dependent on steroid hormones produced by the gonads in response to stimulation by pituitary gonadotropins [13]. In females, 17β-estradiol (E2) regulates the synthesis of hepatic vitellogenins, which are incorporated into the growing oocytes and provide the main nutrient reserves for the developing embryos and prefeeding larvae [14–15]. In males, the androgen 11-ketotestosterone (11KT) is the major steroid that regulates spermatogenesis and plays a critical role in the development of secondary sexual characteristics and induction of reproductive behaviors [16–17]. Because changes in circulating sex steroid levels are correlated with the onset and progression of gametogenesis, they are commonly used as indicators of the stage of maturity in fishes. Seasonal changes in plasma sex steroids have been analyzed in relation to gonadal development in a number of scorpaeniform species [18–20], as well as in other commercially important marine species [21–24]. These studies have enabled the classification of their reproductive cycles into defined maturation periods, and demonstrated that plasma steroid analysis is a useful and relatively non-invasive approach for monitoring reproductive cycles in discrete populations of wild fish. Furthermore, once defined, plasma steroid measures can be used as an important tool for the evaluation of technologies for controlling reproduction of captive fishes in aquaculture.

The current study aimed to characterize the complete reproductive life histories of male and female sablefish. Toward this end, we describe seasonal changes in gonadal macroscopic morphology and histology, and their relationship to plasma sex steroid levels (E2 and 11KT), hepatosomatic and gonadosomatic indices (HSI, GSI), and condition factor (K) in female and male sablefish captured off the Washington coast. We also discuss the utility of these measures as indicators of sexual maturity for fisheries stock assessments, and provide information on basic aspects of sablefish reproductive biology that may facilitate its reproduction in captivity.

## Materials and methods

### Fish collection and sampling

**Fig 1:**
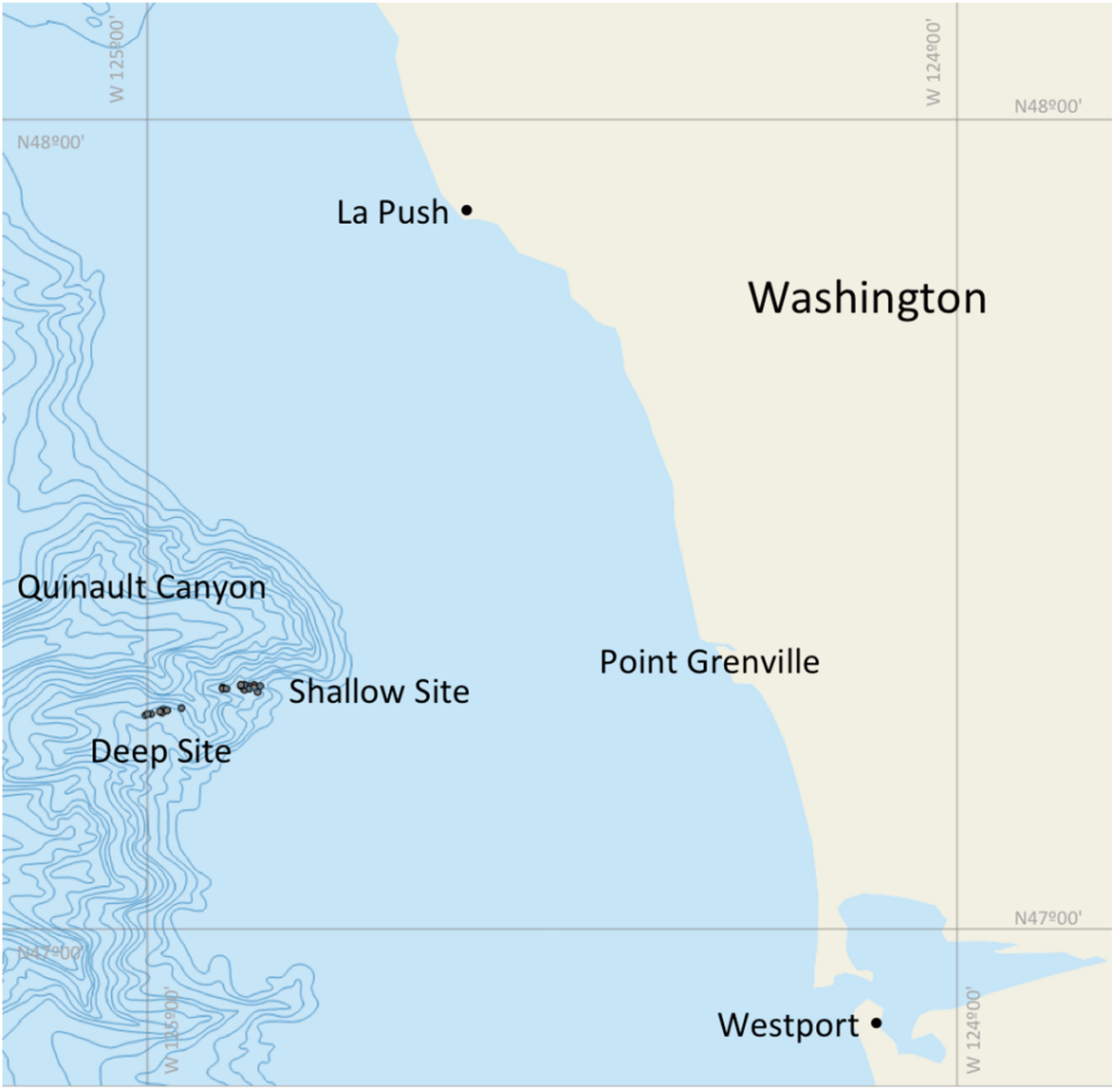
Map of deep and shallow longline sampling sites off the Washington coast over the Quinault Canyon. Sampling was performed 40–50 miles northwest of Gray's Harbor, Westport (WA). See S1 Table for exact details of longline depth and location.

From August 2012 to August 2013, sablefish were collected monthly using demersal longlines in two adjacent areas of the Quinault Canyon, 40–50 miles Northwest of Grays Harbor, WA (Fig 1). Ground-line lengths varied from 600 to 1500 fathoms (1097 to 2743 m). Set depths ranged from 130 to 525 fathoms (238 to 960 m). Details on geographic coordinates and sampling depths are provided in S1 Table. Each longline set was between 1380 and 1840 m long, and each set had 400 to 500 8/0 circle hooks baited with squid and spaced 1.8–3.7 m apart. The hooks used in this study were large enough not to exclude larger fish. In fact, as the results indicate, very large fish that were >70 cm were sampled at times. These hooks are the same ones that are used by commercial long-liners who are frequently targeting larger fish.

The targeted number of fish to be collected per sampling month was at least 15 individuals of each sex. Sampling of fish began in August 2012 at a site on the shelf break that was on average 214 fathoms deep. However, by November, it was not possible to reach the target number of fish at that site. Thus, sampling was moved to an adjacent site that was deeper (average 284 fathoms) and we sampled primarily from this site through the winter until March and April 2013, when test sets at the original site indicated that large numbers of adult fish could again be obtained there. Since the objective of this study was to characterize the reproductive cycle, attempts were made to restrict samples to postpubertal fish. As such, only sablefish above 50 cm in fork length were sampled. Upon capture, fish meeting the length criterion were placed into an aerated fish tote containing seawater, where dissolved oxygen and temperature were monitored (OxyGuard Handy Polaris, Denmark) and adjusted to 80% or greater saturation using an air pump and approximately 4°C using unchlorinated ice. As sablefish do not have swim bladder, they are expected to be less susceptible to barotrauma when brought to the surface.

Once fishing was completed each day, the fish held in the tote were euthanized using cervical dislocation. Total and fork lengths (TL and FL, respectively) to the nearest 5 mm were measured and total body weight (BW) to the nearest 5 g were recorded using a motion compensating scale (Marel M2000 Series, Iceland). Blood (5 ml) was collected from the caudal vasculature using heparinized syringes and placed in glass tubes on crushed ice. Plasma was obtained by centrifugation of whole blood (3000 x g, 15 min, 4°C) and stored at −20°C for sex steroid (E2 and 11KT) analyses by enzyme-linked immunosorbent assays (ELISAs). The head, liver and gonads were removed and stored in separate plastic bags on ice. Once onshore, sagittal otolith pairs were removed from left and right sides of the head and stored in 50% ethanol for age estimation by the break-and-burn method [25]. Gonads and livers were weighed for calculation of the GSI (gonad weight×100/BW) and HSI (liver weight x100/BW), respectively. The length-weight relationship was used to calculate Fulton’s K condition factor (100 BW/FL^3^) [26]. Gonads were photographed using a Nikon D7000 with 18-55mm VR II lens. For histological analyses, two pieces from the middle portion of the gonad were collected and preserved using two different fixatives, so their suitability for histological analyses could be further compared. One piece was preserved in Bouin’s fixative for 48 h before storage in 70% ethanol, while the other was preserved in Davidson´s fixative for 7 days before storage in 70% ethanol.

The study plan and animal care procedures were approved by the NOAA Fisheries Northwest Fisheries Science Center. NOAA Fisheries does not require or provide for review by an Institutional Animal Care and Use Committee for research projects focused solely on fishes. The permit used to sample sablefish was a Scientific Research Permit issued through the NOAA-West coast Region Sustainable Fishery Division. In the present study, fish were euthanized in accordance with guidelines for the euthanasia of finfish of the American Veterinary Medical Association [27].

### Gonadal histology, image analysis and follicle size-frequency analyses

Fixed gonads were dehydrated through a graded series of ethanol, embedded in paraffin wax, sectioned at a thickness of 5 μm, and stained with hematoxylin and eosin. Microscope images were captured using a Nikon (Melville, NY, US) video camera and analyzed with NIS Elements image software version 2.3 (Nikon). As we did not find significant differences between Bouin’s and Davidson’s fixatives, we pursued histological analyses using the former, as used in previous studies [4, 28–29].

After histological examination of the gonad, 12 females and 2 males were identified as prepubertal (see histological criteria in [28, 30]) and thus excluded from the study. Sablefish ovaries from adult females were classified into six stages of development based on histological observations published for this species [6]. Because macroscopic staging schemes are commonly used in surveys and field studies to estimate fish sexual maturity [31], here we also photographed and described the external appearance of the gonads at each stage. Sablefish testes from adult fish were classified into six developmental stages based on histological analyses reported for European sea bass [32]. As for females, a brief description of the external appearance of the testes is provided for each stage of development.

Ovarian follicle size-frequency distributions were determined in sablefish at specific stages of development. For this, ovarian sections from six or ten females were selected for analyses (n = 6 for females at the onset of secondary growth, early, mid and late vitellogenic, and postspawning stages; n = 10 for periovulatory females). The diameter of all intact, cross-sectioned follicles (i.e., those in which the oocyte nucleus was visible in the section) contained in 3.5-mm^2^ frame were measured and counted in 20-μm intervals. To enhance the representation of follicles at each stage, chromatin nucleolus and early perinucleolus-stage oocytes (typically < 80 μm) were not included in the analysis. When the number of cross-sectioned follicles was below 75 counts (normally in late vitellogenesis and periovulatory females), an additional 3.5-mm^2^ frame was analyzed in the same section.

To facilitate the visualization of the results, follicle size-frequency histograms were divided into four categories: perinucleolar, early developing, fully vitellogenic and maturing. (1) perinucleolar (80–160 μm), includes follicles at the late perinucleolus stage; (2) early developing (160–340 μm), includes follicles at the onset of secondary growth (scattered yolk granules, oil droplets and cortical alveoli along the outer margin of the cytoplasm) and early vitellogenesis (cytoplasm partially filled with yolk and lipid globules), these stages were combined in a single category as they typically overlapped in size (see also [6]); (3) fully vitellogenic (340–610 μm), includes follicles with their cytoplasm completely filled with yolk and lipid globules; and (4) maturing (610–760 μm), includes follicles showing signs of germinal vesicle migration and yolk globule fusion.

### Sex steroid analyses

Levels of plasma sex steroids were quantified by ELISA using protocols validated for sablefish [29]. The sensitivities of the assays, calculated as maximum binding minus twice the standard deviation, were 11.9 pg/ml and 4.9 pg/ml for the E2 and 11KT immunoassays, respectively. The ED50s for the E2 and 11KT assays were 1.32 ng/ml and 0.07 ng/ml, respectively. Intra-assay coefficients of variation (CVs; n = 10) were 5.7% and 3.7% for E2 and 11KT, respectively. Inter-assay CVs were 7.3% and 11.2% for E2 and 11KT, respectively. Details on cross-reactivity of E2 and 11KT antibodies, and correlation between levels of E2 and 11KT measured by ELISA and liquid chromatography/tandem mass spectrometry in sablefish plasma samples can be found in [29].

### Statistical analyses

Age, length and weight analyses were conducted in R software version 3.3.0 [33]. Length, weight and age data were analyzed by two-factor ANOVA models where sex and sampling month were used as factors. To accommodate unequal variances among groups, weighted least-squares ANOVA was used and individual observations were weighted by the inverse of the variance of their respective group defined by sex and sampling month. Length, weight and age data were log transformed prior to analysis to normalize the model residuals. Multiple pair-wise comparisons between groups were carried out using the Games-Howell post-hoc test to accommodate unequal sample sizes and variance among groups. For post-hoc comparisons, only un-confounded comparisons (those that differ along a single factor) were carried out. Plasma sex steroid levels, GSI, HSI and K analyses were performed using Prism 6 software (GraphPad Software, Inc., La Jolla, CA). Since sex steroid, GSI, HSI and K data or their log-transformed values did not follow a normal distribution, differences among reproductive stages were analyzed using the non-parametric Kruskal-Wallis H test. Normality was previously tested using the D’Agostino-Pearson omnibus test, while comparisons among groups were carried out using the Dunn’s post-hoc test. Correlation coefficients between reproductive parameters were calculated using Pearson product moment (r).

## Results

### Length, weight and age

**Fig 2:**
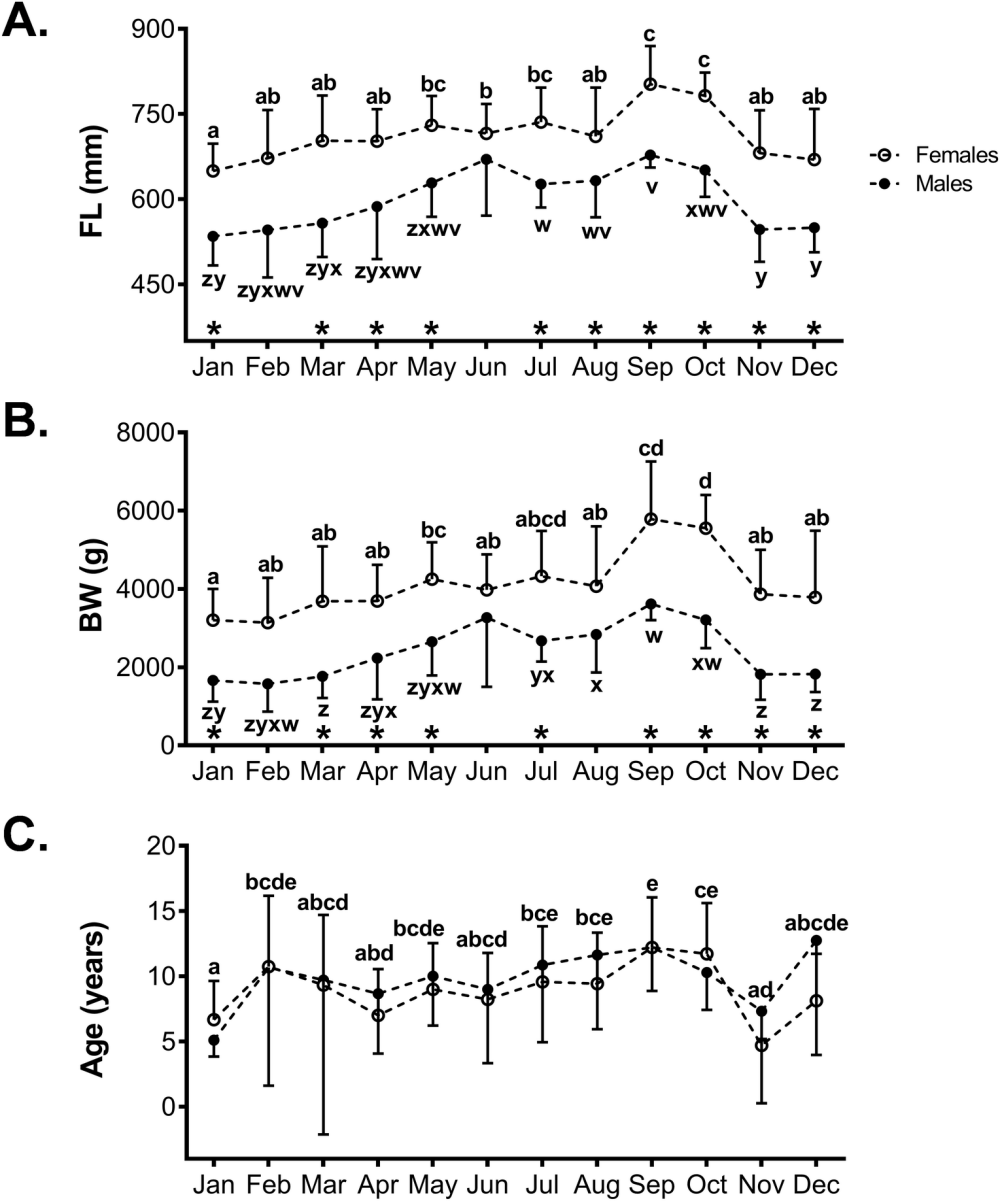
Monthly fork length, body weight and age of sablefish collected off the Washington coast. Fork length (A), body weight (B) and age (C) of female (empty circles) and male (black circles) sablefish. Data are expressed as the mean ± SD. Means not sharing the same letters are significantly different (p<0.05). Male sablefish collected in June were not included in any post-hoc comparisons due to small sample size (n = 2). Asterisks (*) indicate significant differences between males and females within a given month (two-way ANOVA, p<0.05)

Differences in FL, BW and age in female and male sablefish among sampling months are shown in Fig 2. Significant differences in FL were observed between male and female sablefish (F_1,340_ = 310, P <0.0001) and across sampling months (F_11,340_ = 30.084, P <0.0001) (Fig 2A); however, a significant interaction of sex and sampling month was not observed (F_11,340_ = 0.869, P = 0.571). Post-hoc comparisons revealed that the shortest fish were caught during the winter months with lengths increasing until September and October.

A similar pattern was observed for BW, as female sablefish were significantly heavier than males (F_1,340_ = 31.656, P <0.0001) (Fig 2B). While these differences were significant among most sampling months (F_11,340_ = 298.370, P <0.0001), no significant interaction of sex and sampling month was found (F_11,340_ = 1.536, P = 0.117). The lowest body weights were observed during the winter months with a steady increase through September.

The mean age of captured fish was significantly different among sampling months (F_11,340_ = 13.192, P <0.0001), but not different between sexes (F_1,340_ = 0.047, P = 0.828) (Fig 2C). While age differed across sampling months, no clear trends were identified. When the age of fish was examined by month of capture, no significant differences were found in either females or males.

### Stages of ovarian development

#### Onset of secondary growth

**Fig 3:**
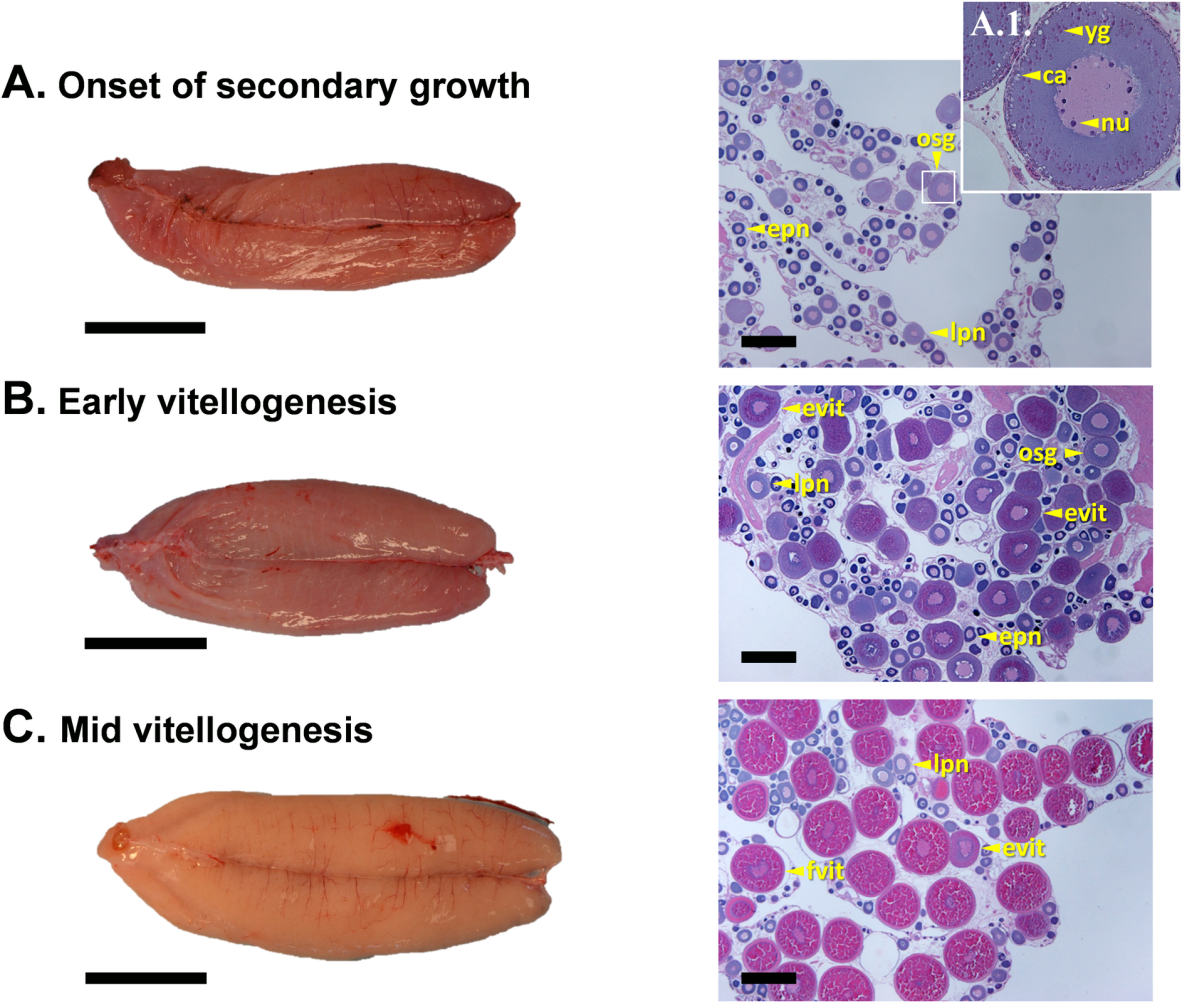
Representative photographs of the external appearance and histological sections of sablefish ovaries at onset of secondary growth, early and mid vitellogenic stages. Onset of secondary growth (A), early vitellogenesis (B) and mid vitellogenesis (C). Detail of an ovarian follicle transitioning to secondary growth is shown in A.1. Abbreviations: yg, yolk granule, ca, cortical alveoli; nu, nucleolus; epn, early perinucleolus follicle; lpn, late perinucleolus follicle; evit, early vitellogenic follicle; fvit, fully vitellogenic follicle. Gonad orientation is anterior portion to the left, posterior to the right. Bars indicate 4 cm in left panels and 500 μm in right panels.

**Fig 4:**
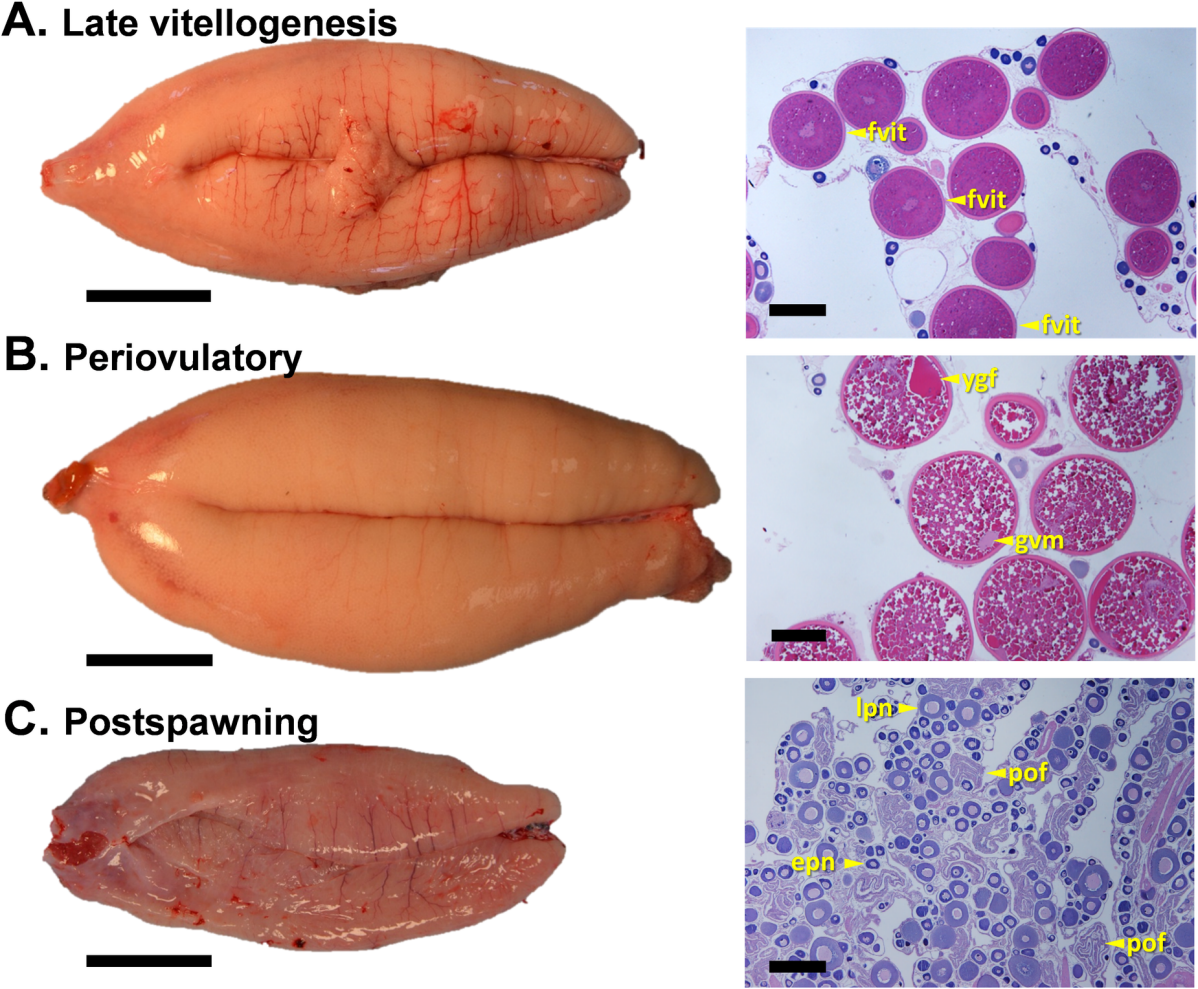
Representative photographs of the external appearance and histological sections of sablefish ovaries at late vitellogenic, periovulatory and postspawning stages. Late vitellogenesis (A), periovulatory (B) and postspawning (C). Abbreviations: epn, early perinucleolus follicle; lpn, late perinucleolus follicle; fvit, fully vitellogenic follicle; gvm, germinal vesicle migration; ygf, yolk granule fusion; pof, postovulatory follicle. Gonad orientation is anterior portion to the left, posterior to the right. Bars indicate 4 cm in left panels and 500 μm in right panels.

**Fig 5:**
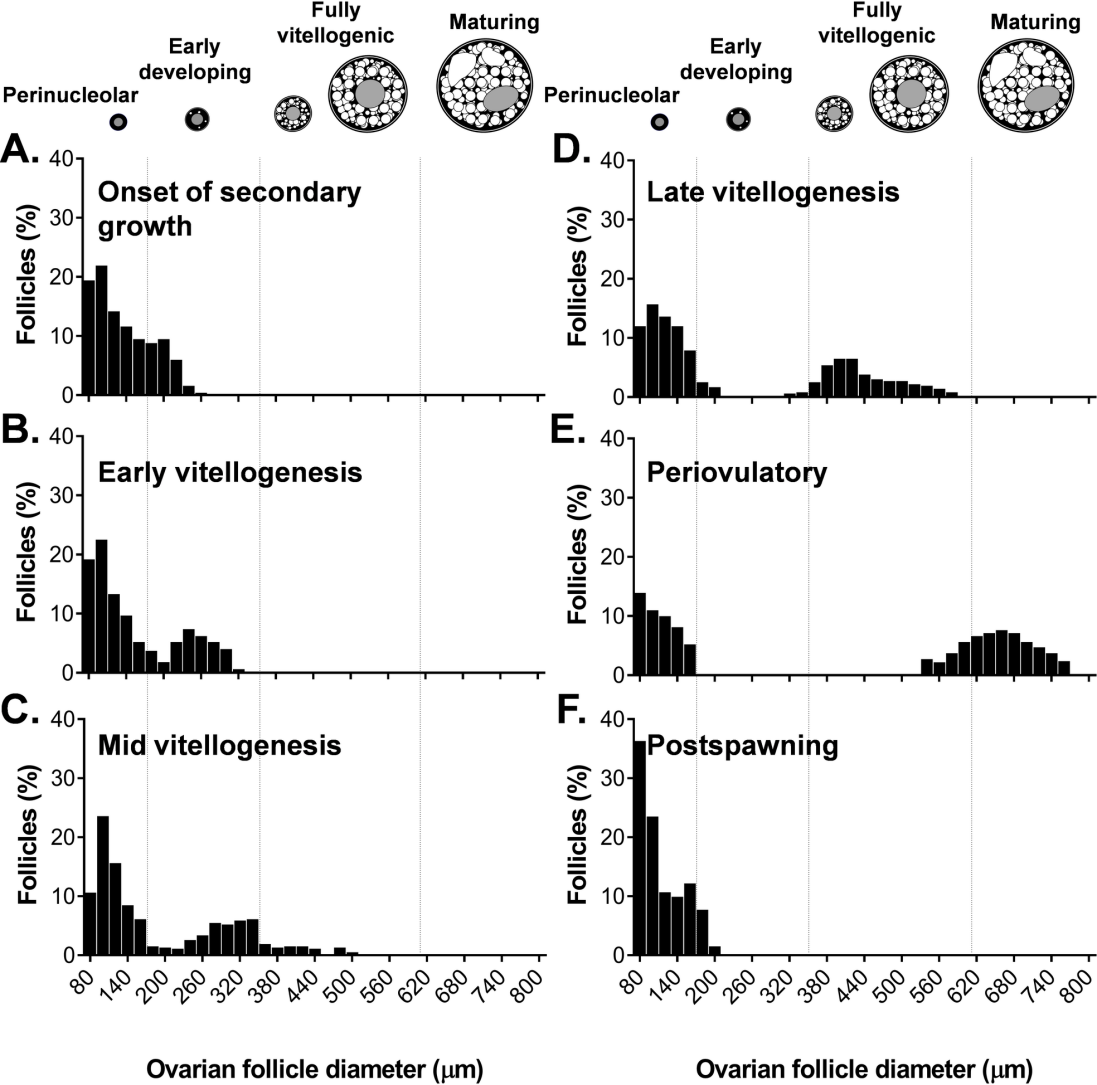
Follicle size-frequency distribution histograms representative of each stage of ovarian development in sablefish. Ovaries at onset of secondary growth (A), early vitellogenesis (B), mid vitellogenesis (C), late vitellogenesis (D), periovulatory (E) and postspawning (F) are shown. A total of 597, 743, 625, 491, 384, 204 and 559 cross-sectioned follicles were measured at these specific stages, respectively. To facilitate the visualization of the results, follicle size-frequency histograms are divided into four categories: perinucleolar, which includes follicles at the late perinucleolus stage (80–160 μm); early developing, which includes follicles at the onset of secondary growth and early vitellogenesis as they typically overlap in size (160–340 μm); fully vitellogenic, which includes follicles with their cytoplasm completely filled with yolk and lipid globules (340–610 μm); and maturing, which includes follicles showing signs of germinal vesicle migration and yolk globule fusion (610–760 μm).

The onset of secondary growth was not accompanied by a major change in the external appearance of the ovaries compared to the prior postspawning stage (Fig 3A, left panel, and Fig 4C left panel). The ovaries at these two stages were similar in color and somewhat flaccid. However, in histological sections, follicles accumulating yolk granules and cortical alveoli along the outer margin of the cytoplasm were visible for the first time (Fig 3A, right panel, and details in Fig 3A.1). Degenerating postovulatory follicles were found at this stage indicating prior spawning events. Perinucleolar and early developing follicles showed a unimodal size-frequency distribution (Fig 5A).

#### Early vitellogenesis

Only slight change in macroscopic morphology was apparent in early vitellogenic ovaries compared to those at onset of secondary growth. While the ovaries at these two stages were similar in color, the ovaries at early vitellogenesis were less flaccid and more rounded (Fig 3B, left panel). Histologically, this stage is characterized by the accumulation of early vitellogenic follicles, in which cytoplasm is partially occupied by yolk granules and lipid globules, but no fully vitellogenic follicles are observed yet. Follicles at onset of secondary growth were still present at this stage. Follicle size-frequency analysis revealed a bimodal distribution for the first time (Fig 5B).

#### Mid vitellogenesis

Ovaries at the mid vitellogenic stage appeared larger relative to the two previous stages and were cream to light orange colored (Fig 3C, left panel). Follicles were discernible with the naked eye in some specimens. Histologically, the most advanced group of follicles had oocytes with the cytoplasm completely filled with yolk globules and lipid inclusions (fully vitellogenic, Fig 3C, right panel) and displayed a substantial increase in size (typically >340 μm, Fig 5C). Early vitellogenic follicles and follicles transitioning to secondary growth were still present in the ovary at this stage (Fig 3C).

#### Late vitellogenesis

Ovaries in the late vitellogenic stage were light orange in color (Fig 4A, left panel); translucent follicles were clearly visible with the naked eye. For the first time, most if not all developing follicles had oocytes with the cytoplasm completely filled by large yolk granules and lipid globules (Fig 4A, right panel). The nucleus was still centrally located and no signs of ovulation were apparent. Size-frequency analysis of the ovarian follicles revealed for the first time a gap between the cohort of vitellogenic follicles and the previtellogenic pool (Fig 5D).

#### Periovulatory

Females at the periovulatory stage had a distinctly swollen and soft abdomen. The ovaries were turgid and voluminous, and orange in color (Fig 4B, left panel). Ovulatory channels were visible with the naked eye, appearing as aqueous ducts running longitudinally along the ovary. Histologically, this stage was characterized by the presence of maturing follicles, in which the nucleus was migrating to the periphery of the cytoplasm (Fig 4B, right panel), and large fully vitellogenic follicles (> 540 μm). For the first time postovulatory and hydrated follicles were observed.

#### Postspawning

Females at the postspawning stage had a soft, distended abdomen. Their ovaries were pink, flaccid, empty and bloodshot in appearance (Fig 4C, left panel), typically containing a small number of unovulated eggs. Histologically, the ovary contained numerous postovulatory follicles as well as perinucleolus stage follicles (Fig 4C, right panel). No early developing or vitellogenic follicles were observed.

### Reproductive cycle of female sablefish from coastal Washington

**Fig 6:**
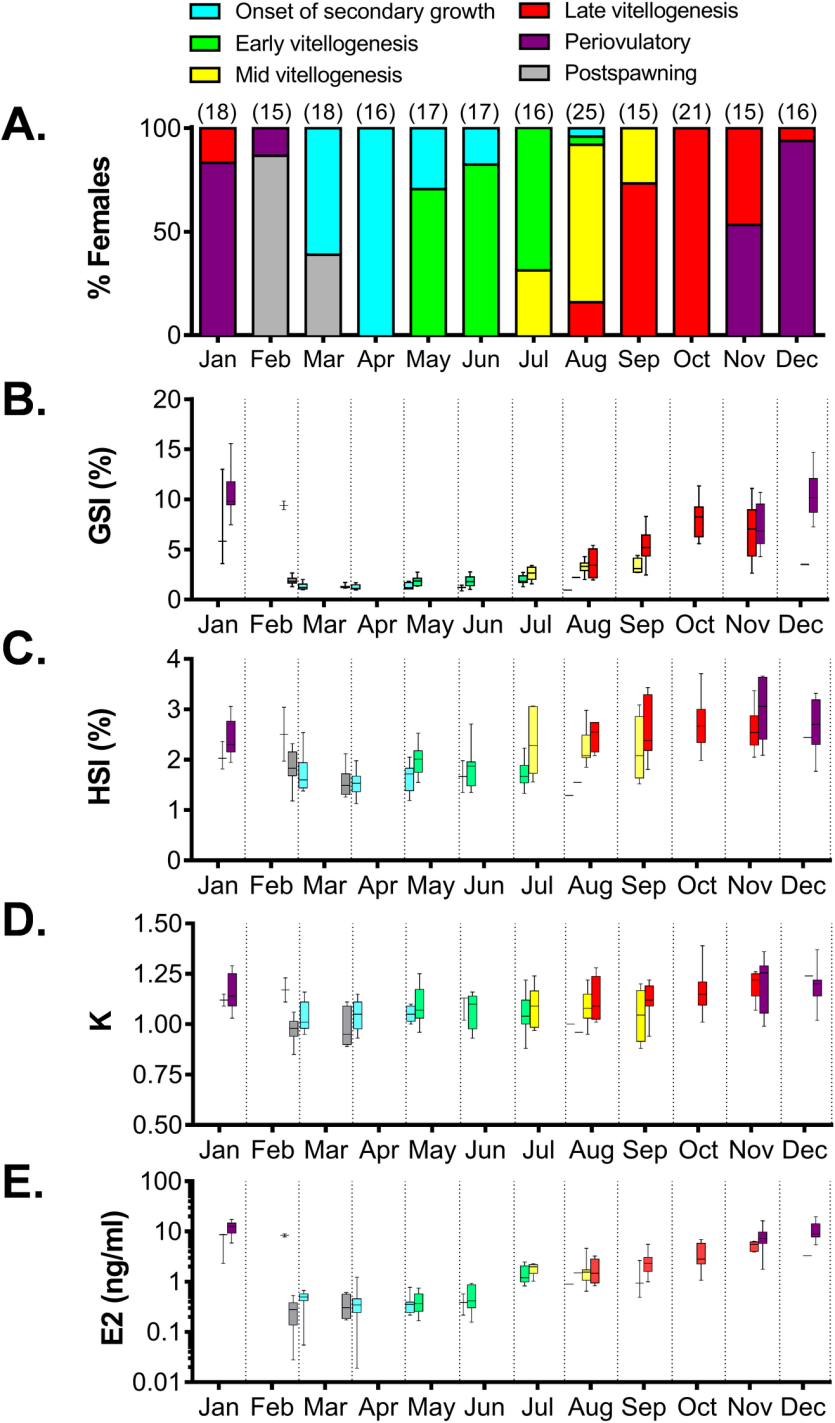
Monthly proportion of female sablefish at specific reproductive stages and their gonadosomatic index, hepatosomatic index, condition factor and plasma 17β-estradiol levels. Proportion of female sablefish at specific reproductive stages (A), gonadosomatic index (B), hepatosomatic index (C), condition factor (D) and plasma 17β-estradiol levels (E). In A, the number of females sampled at each month is indicated in parentheses. In B-E, females were divided into specific stages of ovarian development within a month. In B-E, data are represented by box and whisker plots in which the box extends from the 25th to 75th percentile and the line within the box indicates the median; whiskers are determined by the largest and the smallest values. To improve visualization of lower values, plasma levels of 17β-estradiol are shown in a log10-based scale.

**Fig 7:**
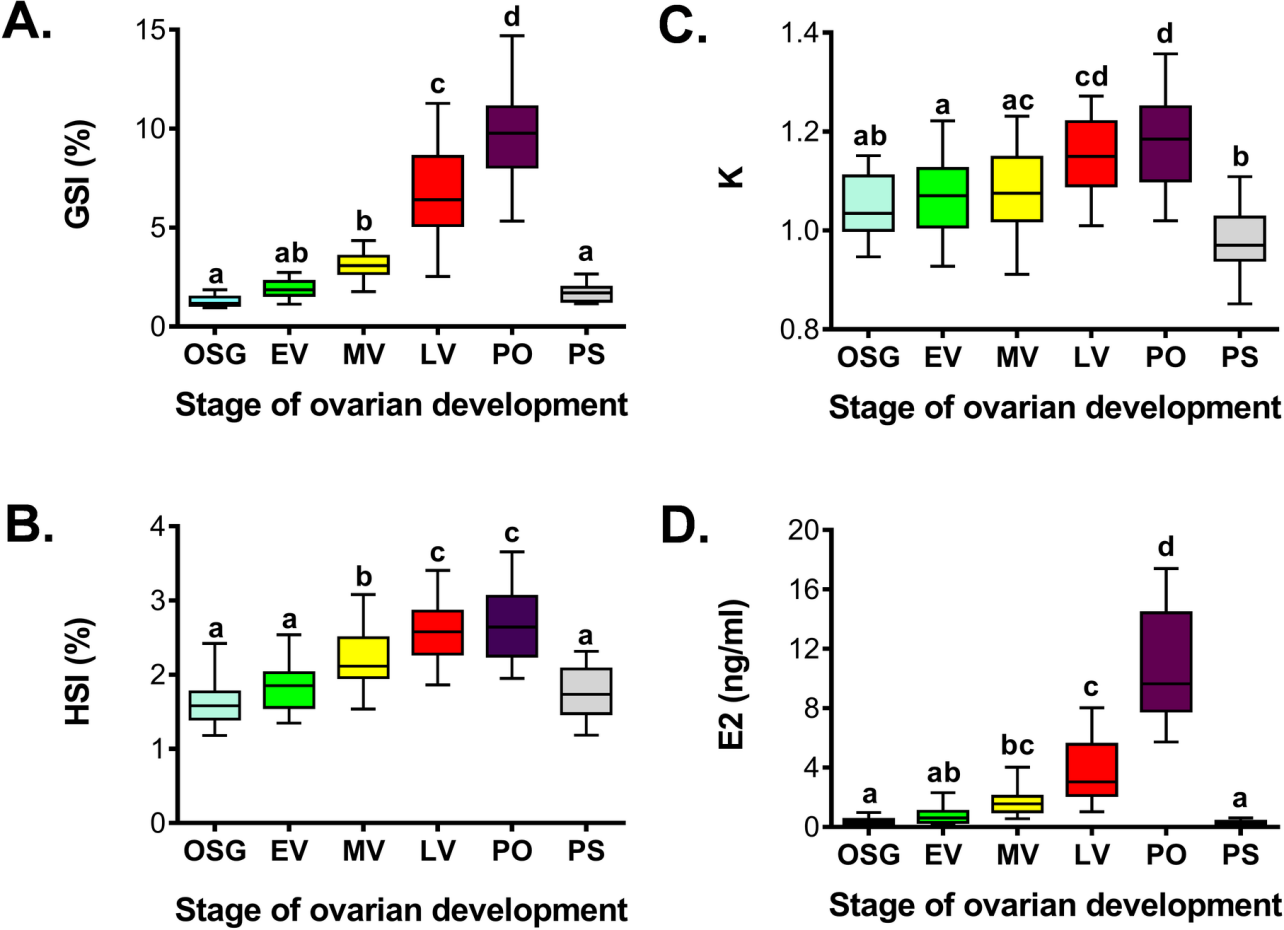
Comparison of gonadosomatic index, hepatosomatic index, condition factor and plasma 17β-estradiol at specific stages of ovarian development in sablefish. Gonadosomatic index (A), hepatosomatic index (B), condition factor (C) and plasma 17β-estradiol (D). The stages of ovarian development include onset of the secondary growth, OSG; early vitellogenesis, EV; mid vitellogenesis, MV; late vitellogenesis, LV; periovulatory, PO; postspawning, PS. Data are represented by box and whisker plots in which the box extends from the 25th to 75th percentile and the line within the box indicates the median; whiskers extend from the 5th to 95th percentile. Shared letters indicate statistical similarities (ANOVA on ranks, p<0.05).

Females at multiple stages of ovarian development were found in most months. Therefore, to show seasonal changes in GSI, HSI, K, and plasma E2 levels throughout the reproductive cycle, these parameters were split by reproductive stage for each sampling month (Fig 6). Furthermore, to permit comparisons among reproductive stages, regardless of sampling month, data were grouped by stage (Fig 7).

Onset of secondary growth was first observed in March (Fig 6A). This transition was only identifiable by histological examination as none of the reproductive parameters analyzed (GSI, HSI, K or E2) changed significantly relative to postspawning females (Fig 7). Vitellogenesis progressed during spring and summer, and was characterized by a steady increase in GSI, HSI and plasma E2 levels (Figs 6 and 7). Late vitellogenic females, in which the recruitment of follicles from the previtellogenic pool has concluded, were first observed in August, and reached their highest proportion (100% of captured females) two months later. Periovulatory females were observed from November to February, with a peak in occurrence in December and January (83 and 93% of captured females, respectively) (Fig 6A). At this stage, GSI, HSI, K and plasma E2 reached maximum levels (Figs 6 and 7). In particular, a sharp surge was observed for the E2 levels, which increased 2.2-fold compared to the previous late vitellogenesis stage (Fig 7D). Post spawning females were found in February and March (Fig 6A), and were characterized by a return to basal GSI, HSI, K and plasma E2 levels (Fig 7). We did not find any histological evidence of skip spawning females (see criteria in [11, 34]), and high intensity atresia (i.e., >25% of ovarian follicles within a section, [6]) was not found in any of the females. Correlation coefficients among reproductive parameters are shown in Table 1. Positive, strong and significant correlation coefficients were found among GSI, HSI, K and plasma E2 during the reproductive cycle of female sablefish.

**Table 1:** Pearson correlation coefficients (r) and p values among reproductive parameters from female (n = 209) and male (n = 159) adult sablefish.

**Females**				
	**E2 (ngml**^**-1**^**)**	**GSI (%)**	**HSI (%)**	**K**
**E2 (ngml^-1^)**		0.785	0.557	0.495
**GSI (%)**	P < 0.0001		0.616	0.567
**HSI (%)**	P < 0.0001	P < 0.001		0.551
**K**	P < 0.0001	P < 0.001	P < 0.001	
**Males**				
	**11KT (ngml**^**-1**^**)**	**GSI (%)**	**HSI (%)**	**K**
**11KT (ngml^-1^)**		0.305	0.263	0.125
**GSI (%)**	P < 0.0001		0.031	0.554
**HSI (%)**	P < 0.001	P > 0.05		0.192
**K**	P > 0.05	P < 0.001	P <0.05	

A summary of the reproductive traits at specific stages of ovarian development is shown in Table 2.

**Table 2:** Summary of reproductive traits at specific stages of ovarian development in sablefish.

Ovarian stage Number of fish at that stage	Reproductive phase based on [35]	Macroscopic examination	GSI (%)	HSI (%)	K	Histological description of the ovary and follicle size distribution	Plasma E2 (ng/ml)
**Onset of secondary growth** N = 36	**Developing**	• Ovaries flaccid and dark pink in color	1.26±0.05 Max: 2.01 Min: 0.86	1.62±0.05 Max: 2.54 Min: 1.13	1.05±0.01 Max: 1.16 Min: 0.93	• First appearance of follicles transitioning to secondary growth (160–280 μm)	0.41±0.04 Max: 1.23 Min: 0.02
**Early vitellogenesis** N = 38	**Developing**	• Ovaries less flaccid and dark pink in color	1.89±0.08 Max: 2.78 Min: 1.02	1.84±0.06 Max: 2.71 Min: 1.33	1.07±0.01 Max: 1.25 Min: 0.88	• Accumulation of early developing follicles (200–320 μm) • Follicles show bimodal distribution	0.78±0.1 Max: 2.47 Min: 0.02
**Mid vitellogenesis** N = 28	**Developing**	• Ovaries lightly swollen and pale cream in color • Few follicles visible	3.13±0.12 Max: 4.39 Min: 1.57	2.25±0.08 Max: 3.09 Min: 1.52	1.08±0.02 Max: 1.24 Min: 0.88	• Accumulation of fully vitellogenic follicles (340–610 μm) • Early developing follicles present	1.73±0.17 Max: 4.62 Min: 0.49
**Late vitellogenesis** N = 47	**Developing**	• Ovaries swollen and light orange in color • Follicles clearly visible	6.77±0.38 Max: 13.01 Min: 1.96	2.60±0.07 Max: 3.71 Min: 1.81	1.15±0.01 Max: 1.39 Min: 0.94	• Most developing follicles are fully vitellogenic • Gap between developing and previtellogenic follicles	3.64±0.30 Max: 8.82 Min: 0.82
**Periovulatory** N = 40	**Spawning capable**	• Swollen and soft abdomen • Ovaries turgid, voluminous and orange in color • Ovulatory channels visible	9.79±0.38 Max: 15.58 Min: 4.29	2.66±0.08 Max: 3.66 Min: 1.77	1.18±0.01 Max: 1.37 Min: 0.99	• Accumulation of maturing follicles (typically > 620μm) • Presence of postovulatory follicles	10.51±0.64 Max: 19.64 Min: 1.77
**Postspawning** N = 20	**Regressing/ Regenerating**	• Ovaries empty and flaccid • Bloodshot in appearance • Unovulated eggs visible	1.70±0.10 Max: 2.66 Min: 1.15	1.74±0.08 Max: 2.32 Min: 1.18	0.98±0.02 Max: 1.11 Min: 0.85	• Presence of postovulatory and atretic follicles • Only perinucleolar follicles are present (<200 μm)	0.30±0.04 Max: 0.61 Min: 0.03

### Stages of testicular development

#### Early recrudescence

**Fig 8:**
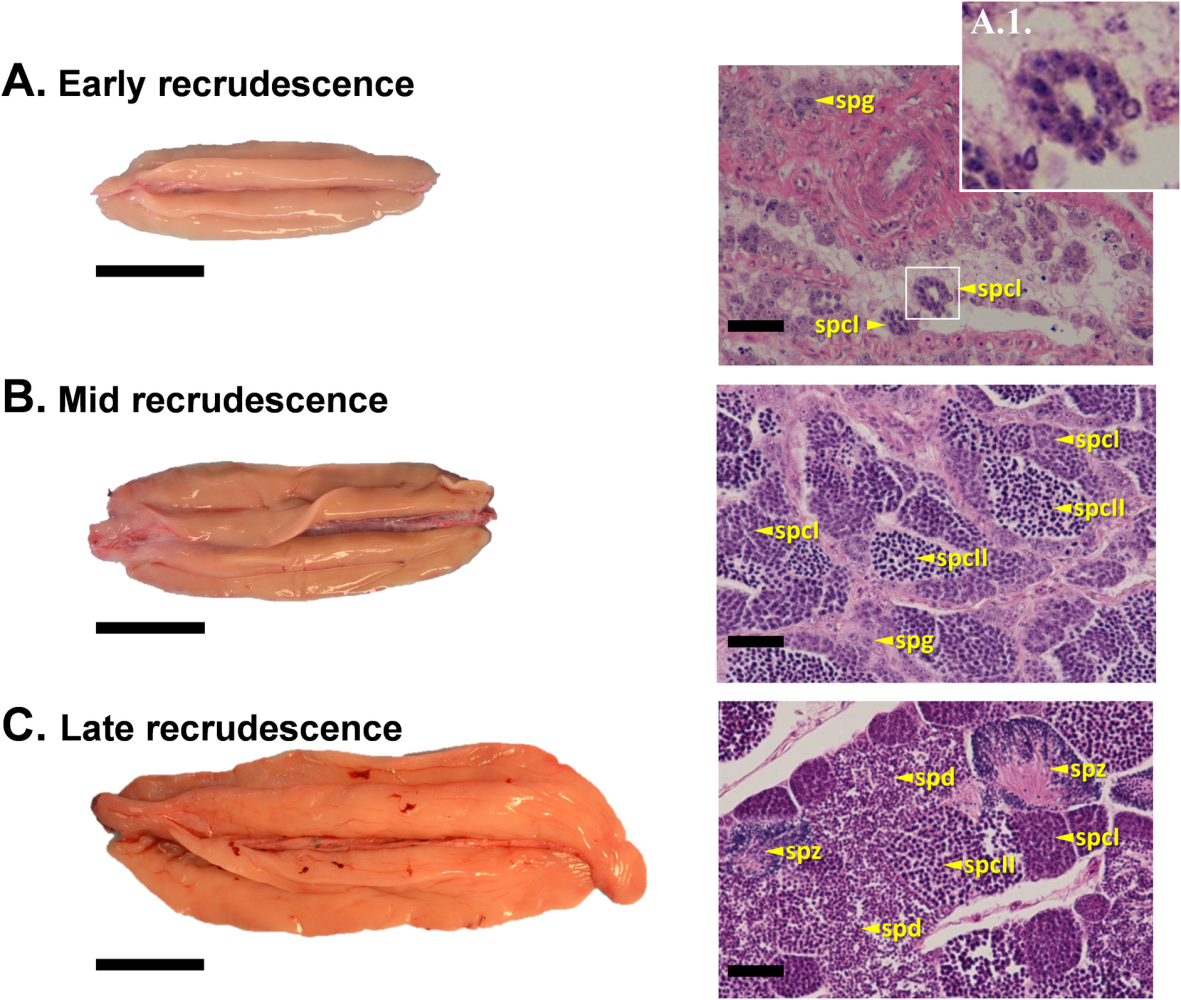
Representative photographs of the external appearance and histological sections of sablefish testes at early, mid and late recrudescence stages. Early recrudescence (A), mid recrudescence (B) and late recrudescence (C). Detail of cyst containing secondary spermatocytes is shown in A.1. Abbreviations: spg, spermatogonia; spcI, primary spermatocyte; spcII, secondary spermatocyte; spd, spermatid; spz, spermatozoa. Gonad orientation is anterior portion to the left, posterior to the right. Bars indicate 4 cm in left panels and 100 μm in right panels.

Testes in early recrudescence were small, thin and cream colored (Fig 8A, left panel). Histologically, this stage was characterized by patches of proliferating spermatogonia and the first appearance of primary spermatocytes which appeared as small, round cells with a basophilic, granular nucleus that was difficult to distinguish from the cytoplasm (Fig 8A and 8A.1). These patches of germ cells were interspersed with regressed testicular tissue from previous spawning.

#### Mid recrudescence

Testes in mid recrudescence were typically thicker and longer, but did not differ in color or general shape from those of the previous stage (Fig 8B, left panel). The folds of testicular tissue appeared more expanded. At this stage, abundant clusters of primary and secondary spermatocytes were present (Fig 8B, right panel), indicating that germ cells thorough the testis had entered meiosis. Secondary spermatocytes were smaller than primary spermatocytes and had a non-granular, highly basophilic nucleus that occupied most of the cytoplasm. Some small cysts containing spermatids were observed; spermatids were similar in appearance to secondary spermatocytes but smaller in size.

#### Late recrudescence

Testes in late recrudescence were turgid and voluminous in appearance (Fig 8C, left panel). Clutches of spermatids had initiated spermiogenesis, and the first accumulation of spermatozoa was observed (Fig 8C, right panel). Spermatozoa were characterized by small basophilic, rounded heads and large acidophilic flagella. At this stage, all types of male germ cells could be identified.

#### Spermiating

**Fig 9:**
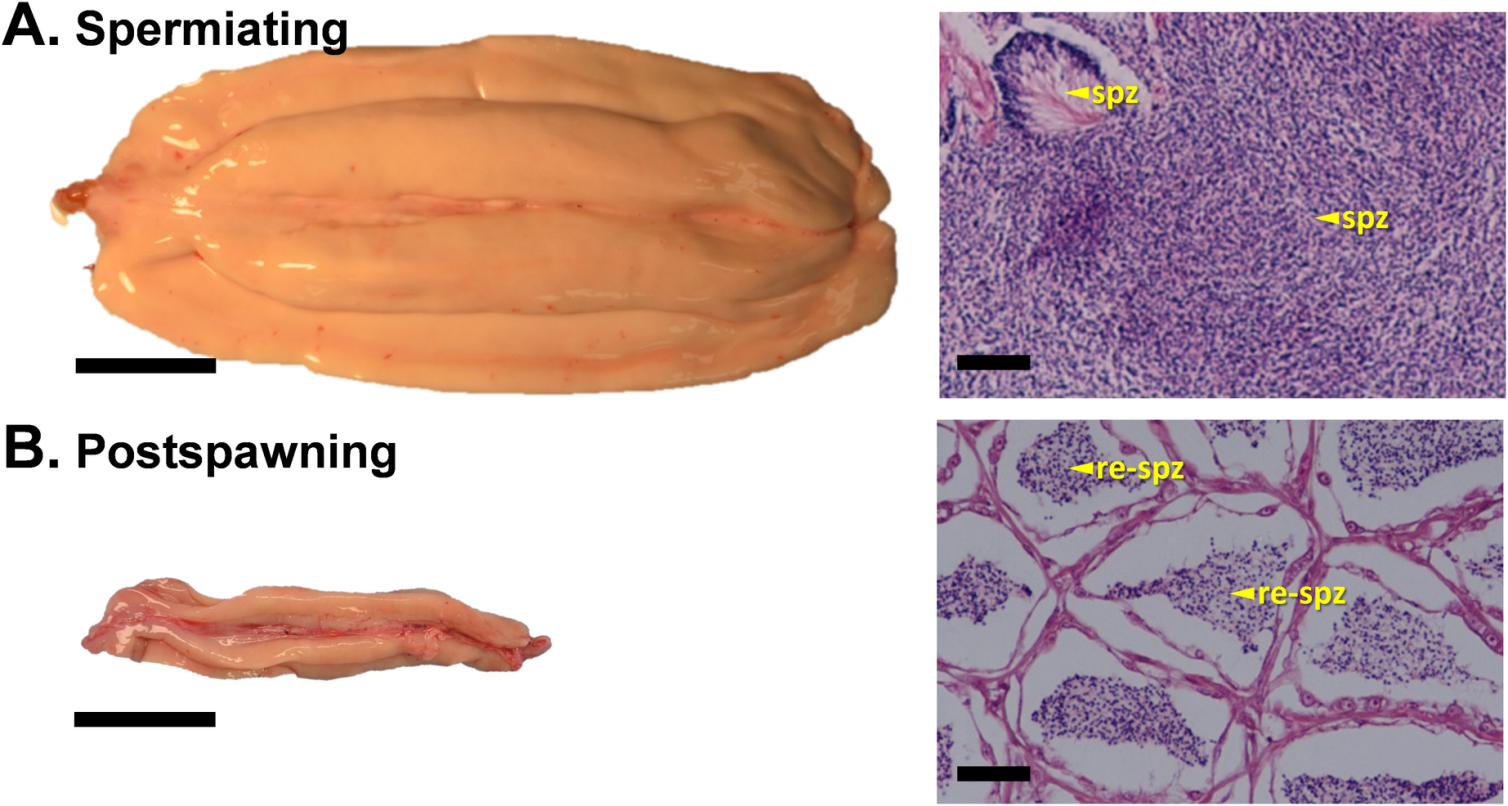
Representative photographs of the external appearance and histological sections of sablefish testes at spermiating and postspawning stages. Spermiating (A) and postspawning (B). Abbreviations: spz, spermatozoa; re-spz, residual spermatozoa. Gonad orientation is anterior portion to the left, posterior to the right. Bars indicate 4 cm in left panels and 100 μm in right panels.

Testes from spermiating males reached their maximum size (Fig 9A, left panel) and copious amounts of milt were released from them with no pressure applied. Folds of testicular tissue were less flattened compared to earlier stages and the testis was lighter in color. After completion of spermiogenesis, the cyst walls had opened and massive pools of spermatozoa were observed via histology (Fig 9A, right panel). While some small cysts of spermatids were still present, spermatocytes were not observed at this stage.

#### Postspawning

Testes from postspawning males were darker in color, flaccid and shrunken in size (Fig 9B, left panel) compared to other stages. Lobules of the testes were smaller and interlobular tissue had become noticeably thicker (Fig 9B, right panel). The folds of testicular tissue were much flatter compared to early recrudescence. Spent testes had numerous nearly empty ducts with small amounts of residual sperm. No spermatocytes or spermatids were observed.

### Reproductive cycle of male sablefish from coastal Washington

**Fig 10:**
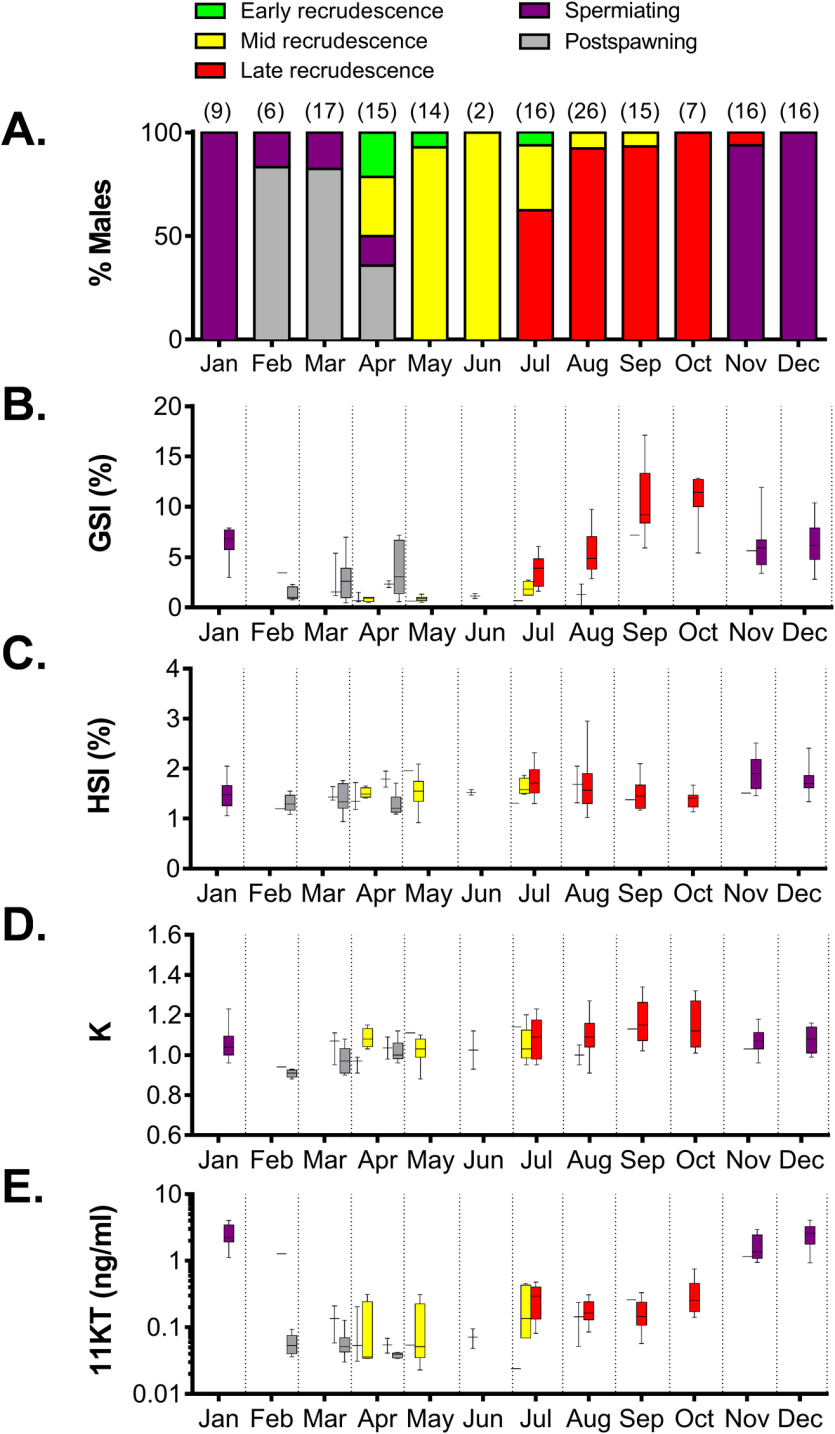
Monthly proportion of male sablefish at specific reproductive stages and their gonadosomatic index, hepatosomatic index, condition factor and 11-ketotestosterone levels. Proportion of male sablefish at specific reproductive stages (A), gonadosomatic index (B), hepatosomatic index (C), condition factor (D) and 11-ketotestosterone levels (E). In A, the number of males sampled at each month is indicated in parentheses. In B-E, males were divided into specific stages of testicular development within a month. In B-E, data are represented by box and whisker plots in which the box extends from the 25th to 75th percentile and the line within the box indicates the median; whiskers are determined by the largest and the smallest values. In order to improve visualization of lower values, plasma levels of 11-ketotestosterone are shown in a log10-based scale.

**Fig 11:**
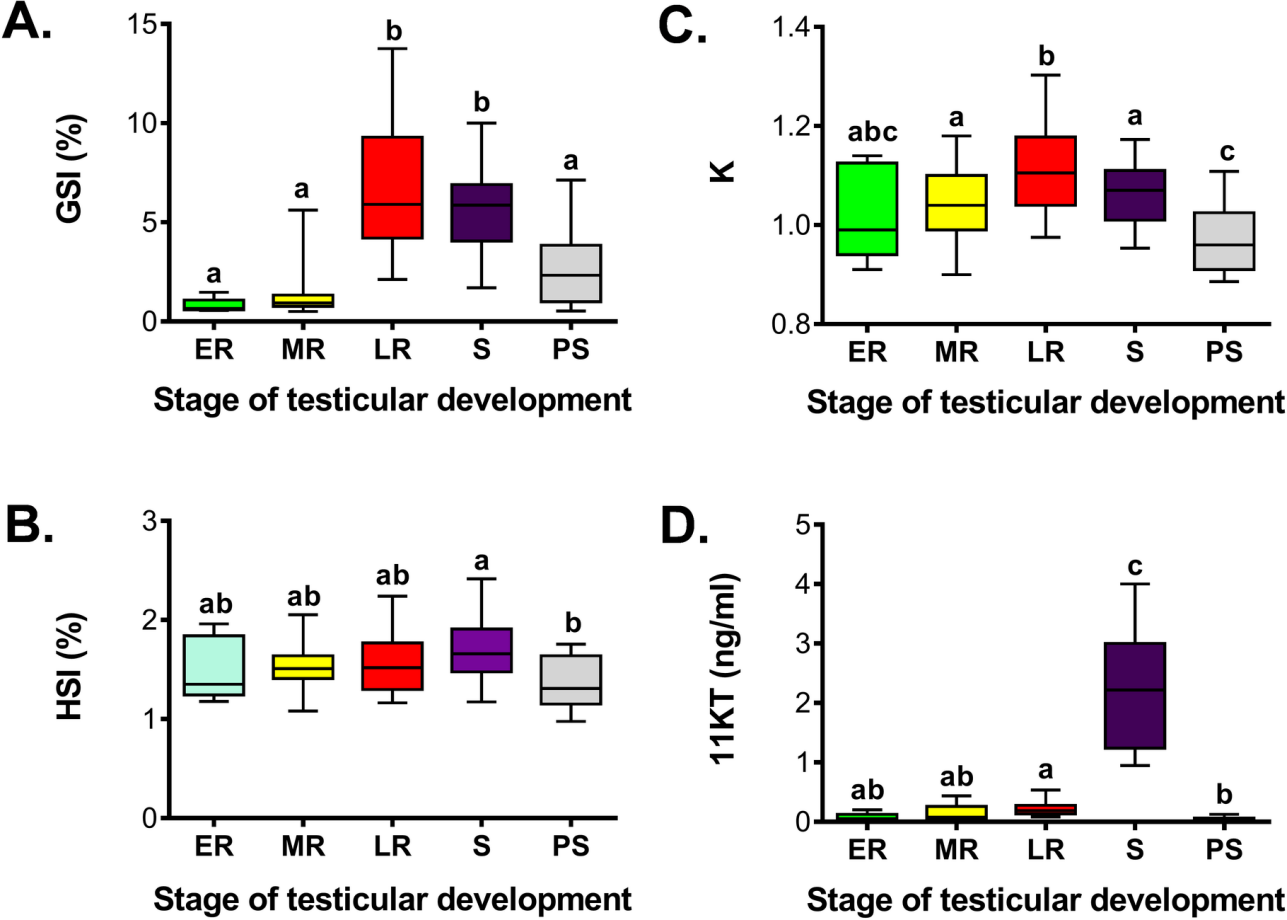
Comparison of gonadosomatic index, hepatosomatic index, condition factor and plasma 11-ketotestosterone at specific stages of testicular development in male sablefish. Gonadosomatic index (A), hepatosomatic index (B), condition factor (C) and plasma 11-ketotestosterone (D). Stages of testicular development include early recrudescence, ER; mid recrudescence, MR; late recrudescence, LR; spermiating, S; postspawning, PS. Data are represented by box and whisker plots in which the box extends from the 25th to 75th percentile and the line within the box indicates the median; whiskers extend from the 5th to 95th percentile. Shared letters indicate statistical similarities (ANOVA on ranks, p<0.05).

Because males at multiple reproductive stages were observed in most months, GSI, HSI, K and plasma 11KT levels were split into stages for each specific month as done with females (Fig 10). To permit comparisons among reproductive stages, regardless of sampling month, data were also grouped by stage (Fig 11).

The first evidence of early testicular recrudescence was observed in April (Fig 10A). However, this stage could only be unambiguously identified by histological analysis, as the macroscopic morphology of the testis, GSI, HSI, K and plasma 11KT levels did not vary significantly relative to the postspawning stage. Testicular recrudescence proceeded during spring and summer, a period during which all types of germ cells were observed. The first clusters of spermatozoa appeared in males caught in summer, during July, indicating the onset of late recrudescence. At this stage, males showed a significant increase in GSI (5.2-fold higher than the previous mid recrudescence stage) and K, while plasma levels of 11KT remained low (Fig 11D). Spermiating males were observed from November to April, with a peak in their occurrence in December and January (100% of captured males). At this stage, a sharp increase in plasma 11KT was observed (9.3-fold higher than the previous late recrudescence stage, Fig 11D). Postspawning males were caught from February to April, and were characterized by a significant decrease in all four reproductive parameters (Figs 10 and 11). We did not find any evidence of skip spawning males (see criteria in [34]). Correlation coefficients among reproductive parameters are shown in Table 1. Positive and significant correlation coefficients during the reproductive cycle of male sablefish were found, being the most significant for GSI and 11KT, GSI and K and 11KT and HSI.

A summary of the reproductive traits at specific stages of testicular development is shown in Table 3.

**Table 3:** Summary of reproductive traits at specific stages of testicular development in sablefish.

Testicular stage Number of fish at that stage	Reproductive phase based on [35]	Macroscopic examination	GSI (%)	HSI (%)	K	Histological description of the testis	Plasma 11KT (ng/ml)
**Early recrudescence** N = 5	**Developing**	• Testes thin, smooth and uniformly textured. Pale cream in color	0.80±0.17 Max: 1.47 Min: 0.56	1.51±0.14 Max: 1.96 Min: 1.18	1.02±0.04 Max: 1.14 Min: 0.91	• Germinal cysts containing spermatogonia • Cysts containing primary spermatocytes	0.07±0.03 Max: 0.20 Min: 0.02
**Mid recrudescence** N = 27	**Developing**	• Testes thicker and longer. Pale cream in color	1.32±0.25 Max: 7.19 Min: 0.18	1.56±0.05 Max: 2.09 Min: 0.92	1.04±0.01 Max: 1.20 Min: 0.88	• Abundant cysts containing primary and secondary spermatocytes • Few cysts containing spermatids	0.14±0.03 Max: 0.45 Min: 0.02
**Late recrudescence** N = 56	**Developing**	• Testes voluminous and lighter in color • Milt cannot be extruded	7.06±0.49 Max: 17.11 Min: 1.62	1.58±0.05 Max: 2.95 Min: 1.02	1.11±0.01 Max: 1.34 Min: 0.91	• All types of germinal cells • Accumulation of spermatozoa	0.23±0.02 Max: 1.16 Min: 0.06
**Spermiating** N = 46	**Spawning capable**	• Milt released after gentle pressure in abdomen • Testes greatly enlarged and light cream in color • Testes milk easily	5.78±0.34 Max: 11.91 Min: 1.17	1.73±0.05 Max: 2.51 Min: 1.06	1.06±0.01 Max: 1.23 Min: 0.94	• No primary or secondary spermatocytes • Small populations of spermatids • Massive accumulation of spermatozoa	1.96±0.17 Max: 4.06 Min: 0.05
**Postspawning** N = 25	**Regressing/ Regenerating**	• Testes thin and smooth • Some milt release after compression	2.64±0.4 Max: 7.14 Min: 0.48	1.36±0.05 Max: 1.76 Min: 0.94	0.97±0.01 Max: 1.12 Min: 0.88	• Lobules shrink and interlobular tissue thickened • No germinal cells • Residual spermatozoa	0.05±0.03 Max: 0.13 Min: 0.03

## Discussion

This is the first complete report of the annual reproductive life history of female and male sablefish. Through macroscopic, histological and hormonal analyses we provide a detailed picture of the reproductive cycle of sablefish from the U.S. Washington coast, indicating the chronology and major stages of gametogenesis, and spawning period. The results presented here provide the foundation for comparative studies into the reproductive biology of this species across its range.

We sampled a total of 209 female and 159 male adult sablefish caught by longline monthly through the course of a year. Overall, females were significantly larger and heavier than males, which is consistent with other studies in sablefish [3, 36–38]. Significant differences in size and body weight were also observed among the sampling months. The smallest fish were caught in November, December and January, and the largest fish caught in September and October. This is consistent with early studies of the sablefish fishery that suggest seasonal changes in the depth distribution of sablefish may occur where larger fish move into deeper waters in the winter [39–40].

The first signs of ovarian follicle recruitment into secondary growth were observed in females caught in March. This transition was characterized by the appearance of yolk granules and some small cortical alveoli along the outer margin of the cytoplasm. Formation of cortical alveoli simultaneously or right before of the incorporation of vitellogenins has been reported in other iteroparous species [41–42]. Females at this early developmental stage were only found in a brief period of the year (March to June, with the exception of one female caught in August), suggesting that, at least in this location, once females start the transition to secondary growth they will typically continue through vitellogenesis and spawning that season. This situation contrasts with species where oocytes can remain inactive at the cortical alveolus stage for an extended period of time, even years [43–44]. Therefore, our finding has potential applications for fish stock assessment purposes, as female sablefish at this early stage of development can be categorized as developing [35], and are likely to spawn the next spawning cycle.

Size-frequency analyses of ovarian follicles revealed a group-synchronous recruitment of oocytes. Further, the resulting bimodal size distribution found in sablefish is characteristic of determinate spawners, wherein fecundity is determined before the onset of the spawning season [45]. Females at the late vitellogenesis stage showed for the first time a major gap between the cohort of developing follicles and the previtellogenic pool (>150 μm, see Fig 5D), suggesting that at this stage the recruitment of follicles has ceased. Therefore, based on these findings, the annual recruitment of ovarian follicles in sablefish likely starts in early spring and is completed by fall, 2–4 months prior to the spawning season.

The fact that in late vitellogenic females the recruitment of ovarian follicles is already completed and ovulation has not started yet, makes these females valuable for annual fecundity estimates. If a female is sampled too early (i.e., mid vitellogenesis or earlier), the batch of developing follicles will not be completely separated from the previtellogenic pool, and consequently estimates may be imprecise. By contrast, if a female is sampled too late (i.e., periovulatory), spawning may have begun and the stock of advanced follicles reduced, thereby leading to an underestimate of total fecundity. In sablefish this could be significant, since a single spawning event may result in the release of up to a third of the standing stock of maturing follicles [10]. Therefore, considering the low incidence of ovarian atresia found in this study and by others [6, 9], and based on the high proportion of late vitellogenic females in September/ October, it seems that early fall is an optimal time frame to estimate annual fecundity in sablefish, at least in this location.

A better understanding of the reproductive biology of male fishes is key to the development of sound management strategies for commercially exploited stocks [46]. However, relative to females, little attention is generally paid to male reproductive traits in stock assessment studies and in particular, no studies had previously examined the reproductive life history of male sablefish. We found that in coastal Washington, early testis recrudescence takes place as early as April, as indicated in our study by the first appearance of primary spermatocytes [35, 47]. However, it is likely that a period of rapid proliferation of spermatogonia towards meiosis, which precedes the appearance of primary spermatocytes and determines the onset of spermatogenesis in fishes [17], may have happened in earlier months. Interestingly, the low incidence of males at the early recrudescence stage suggests that primary spermatocytes may transition quickly to the first meiotic division giving rise to secondary spermatocytes, typically found in mid and late recrudescence stages.

Males at the late recrudescence stage exhibited all types of germ cells and were characterized by a massive increase in GSI. It is worth noting that we recorded a GSI of 17% for an 11-year-old male (3.7 kg, captured in September 2012). While these GSIs are unusually high compared to other fishes, similar GSIs were reported for winter flounder (10%, [24]) and Atlantic cod (9–10%, [47]). In teleosts, testicular weight gain during spermatogenesis is attributable mainly to germ and Sertoli cell proliferation [17, 48]. Hence, our findings suggest a maximum activity of germ cell recruitment and development towards spermatogenesis in males at the late recrudescence stage. Later on, during spermiogenesis, the absence of spermatocytes and the gradual decrease of spermatids suggest that male germ cell recruitment has concluded. The significant decrease in GSI during spermiation is likely a result of the morphological changes associated with the differentiation of spermatids into spermatozoa [17, 49]; these changes may include flagellum formation, nuclear condensation, and elimination of organelles and cytoplasm. Additionally, the decrease in GSI in spermiating males could be due in part to a loss of milt while fish were being brought to the surface. This was not assessed in this study but it is a possibility given the dramatic change in pressure.

Histological evidences as well as the generalized decrease in GSI (in both females and males) in February suggest that sablefish in coastal Washington primarily spawn in winter, during January and February. The reproductive timing of sablefish from California to Canada, and even in the western Pacific, has been addressed in several prior studies. In many of these studies, the period during which sablefish "spawn" has been broadly defined as occurring from as early as October to November [10, 50–51] to as late as March [5, 51]. However, in some of these studies it was unclear what “spawning” actually referred to. If spawning is defined as the time during which gametes are released and fertilization takes place, it seems that this period may actually be much shorter and may be similar across the range of this species. The precise time of spawning (as defined above) is difficult to determine from males since milt can be expressed from males over a protracted period, and generally males are reproductively active for longer periods than females. In contrast, spawning can be more accurately determined from females by assessing the decrease in GSI or the presence/absence of hydrated oocytes and postovulatory follicles. When these criteria are used, however, it appears that spawning in sablefish occurs primarily between January and February at least off the Canadian and Washington coast ([38]; present study). Likewise, Hunter et al. [10] indicated that the size of ovarian follicles in sablefish caught off central California increased in the fall and reached a size in January that was consistent with the onset of hydration, suggesting that spawning occurred then. The occurrence of females that contain fully developed ovaries in December seems to be consistent across studies regardless of the way samples were assessed. This is even true for females in the western North Pacific [12]. Thus it might seem reasonable to hypothesize that the peak time when spawning (release of gametes) occurs is in winter, during January-February, across the range of sablefish. However, the timing of spawning might also be influenced by the depth at which fish reside, the age of the fish or their precise location. For example, surveys on seamounts have indicated that sablefish are "ripe, spawning or recently spent" in July [52]. In this scenario, female and male sablefish would presumably undergo late vitellogenesis and late recrudescence, respectively, in the spring rather than fall.

To better understand the role of sex steroids during gonadal development in sablefish, we examined the seasonal profile of plasma E2 and 11KT. In females, vitellogenic growth was characterized by an increase in E2 levels, which occurred in parallel with rising GSI and HSI values. The positive and strong correlation between E2 and HSI/GSI found in this study supports the regulatory role of E2 in the hepatic synthesis of vitellogenin, prior to its incorporation into developing ovarian follicles [14, 53]. It is interesting to note, however, that maximal levels of E2 were reached in periovulatory females. Consistent with this, peak levels of E2 around or during the spawning period have been reported in a number of multiple batch spawners [23, 54–55], and it is generally accepted that this reflects the recruitment of the least advanced vitellogenic oocytes into final maturation.

In male fishes, 11KT is considered the most important androgen [16]. 11KT plays a physiologically important role in initiating spermatogenesis. In the Japanese eel and African catfish, it is well characterized that 11KT acts directly on the testes to stimulate early mitotic divisions of spermatogonia and surrounding somatic cells [56–57]. In sablefish, the transition through early stages of spermatogenesis was characterized by low 11KT levels (<0.5 ng/ml), while a sharp increase was observed in maturing males, coinciding with the presence of free spermatozoa in the lumen. A similar temporal profile was reported in Atlantic halibut [58], Atlantic cod [23] and Japanese sardine [59], supporting the idea that 11KT has a role in spermiation, development of secondary sexual characters or reproductive behavior in fishes [16].

In conclusion, we characterized for the first time the complete reproductive cycle of sablefish, providing baseline data for the maturity schedule of this species. Ovarian development in sablefish is characterized by a group-synchronous recruitment of oocytes, which starts in March, and is completed by fall, 2–4 months prior to the spawning season. In males, germ cell recruitment and development starts in April, and reaches its highest activity during fall. We found periovulatory females and spermiating males as early as November, however, it seems likely that sablefish in coastal Washington primarily spawn in winter, during January and February. While histological and GSI analyses provide univocal information on the reproductive stage of the fish, we found that plasma sex steroids may be an appropriate non-lethal alternative to identify sablefish undergoing late stages of gametogenesis prior to final maturation. Further studies of the changes in pituitary gonadotropins during the reproductive cycle may provide endocrine indices of early stages of gametogenesis in this species.

## Supporting information

S1 Table Date, fishing midpoint geographic coordinates and depths at the deep and shallow sampling sites during 2012 and 2013. (DOCX)
