# Supplementary material for: Reproductive life history of sablefish (Anoplopoma fimbria) from the U.S. Washington coast
Source: PLoS One. 2017 Sep 8;12(9):e0184413. doi: 10.1371/journal.pone.0184413 (PMC5590928; doi:10.1371/journal.pone.0184413)
Supplement: S1 Table — (DOCX) [file pone.0184413.s001.docx]

**S1 Table. Date, fishing midpoint geographic coordinates and depths at the deep and shallow sampling sites during 2012 and 2013**.

| Date | Latitude | Longitude | Depth (fa.) | Deep | Shallow |
| --- | --- | --- | --- | --- | --- |
| 8/29/2012 | N 47º17.631' | W 124º52.7705' | 177 |  | X |
| 9/25/2012 | N 47º17.973' | W 124º52.1' | 157 |  | X |
| 9/25/2012 | N 47º17.9015' | W 124º52.508' | 147 |  | X |
| 10/25/2012 | N 47º17.915' | W 124º51.6445' | 160 |  | X |
| 10/25/2012 | N 47º17.7495' | W 124º52.4555' | 157 |  | X |
| 11/26/2012 | N 47º17.45' | W 124º51.8225' | 227 |  | X |
| 11/26/2012 | N 47º17.83' | W 124º52.1045' | 161 |  | X |
| 12/30/2012 | N 47º16.273' | W 124º57.555' | 388 | X |  |
| 1/14/2013 | N 47º16.1945' | W 124º58.839' | 291 | X |  |
| 1/14/2013 | N 47º15.8145' | W 124º59.7465' | 256 | X |  |
| 2/14/2013 | N 47º15.749' | W 125º00.19' | 276 | X |  |
| 2/14/2013 | N 47º16.0185' | W 124º58.946' | 265 | X |  |
| 3/27/2013 | N 47º18.012' | W 124º52.819' | 172 |  | X |
| 3/27/2013 | N 47º15.967' | W 124º58.91' | 254 | X |  |
| 4/24/2013 | N 47º18.009' | W 124º52.904' | 190 |  | X |
| 4/24/2013 | N 47º16.0055' | W 124º59.0865' | 271 | X |  |
| 5/29/2013 | N 47º17.972' | W 124º52.8135' | 172 |  | X |
| 5/29/2013 | N 47º17.823' | W 124º54.4655' | 322 |  | X |
| 6/26/2013 | N 47º17.76' | W 124º54.193' | 286 |  | X |
| 6/26/2013 | N 47º17.919' | W 124º53.1215' | 182 |  | X |
| 7/24/2013 | N 47º17.7115' | W 124º54.401' | 325 |  | X |
| 7/24/2013 | N 47º17.9885' | W 124º53.1035' | 204 |  | X |
| 8/28/2013 | N 47º17.7115' | W 124º54.401' | 325 |  | X |
| 8/28/2013 | N 47º17.9885' | W 124º53.1035' | 204 |  | X |
| 11/21/2013 | N 47º15.831' | W 125º00' | 280 | X |  |
| 11/21/2013 | N 47º16.1245' | W 124º58.5745' | 281 | X |  |
| 12/19/2013 | N 47º16.1245' | W 124º58.5745' | 281 | X |  |
| 12/19/2013 | N 47º17.76' | W 124º54.193' | 281 |  | X |
